# Glycocalyx Components Detune the Cellular Uptake of Gold Nanoparticles in a Size- and Charge-Dependent Manner

**DOI:** 10.1021/acsabm.2c00595

**Published:** 2022-10-14

**Authors:** Beatrix Peter, Nicolett Kanyo, Kinga Dora Kovacs, Viktor Kovács, Inna Szekacs, Béla Pécz, Kinga Molnár, Hideyuki Nakanishi, Istvan Lagzi, Robert Horvath

**Affiliations:** † Nanobiosensorics Laboratory, Institute of Technical Physics and Materials Science, Centre for Energy Research, Konkoly-Thege út 29-33, BudapestH-1120, Hungary; ‡ Department of Biological Physics, Eötvös University, BudapestH 1117, Hungary; § Thin Films Laboratory, Institute of Technical Physics and Materials Science, Centre for Energy Research, Konkoly-Thege út 29-33, BudapestH-1120, Hungary; ∥ Department of Anatomy, Cell and Developmental Biology, ELTE, Eötvös Loránd University, Pázmány Péter Stny. 1/C, BudapestH-1117, Hungary; ⊥ Department of Macromolecular Science and Engineering, Graduate School of Science and Technology, Kyoto Institute of Technology, Matsugasaki, Kyoto606-8585, Japan; # Department of Physics, Institute of Physics, Budapest University of Technology and Economics, Műegyetem Rkp. 3, BudapestH-1111, Hungary; ¶ ELKH-BME Condensed Matter Research Group, Műegyetem Rkp. 3, BudapestH-1111, Hungary

**Keywords:** gold nanoparticles, glycocalyx, enzyme digestion, cell adhesion, RWG biosensor, cellular uptake kinetics

## Abstract

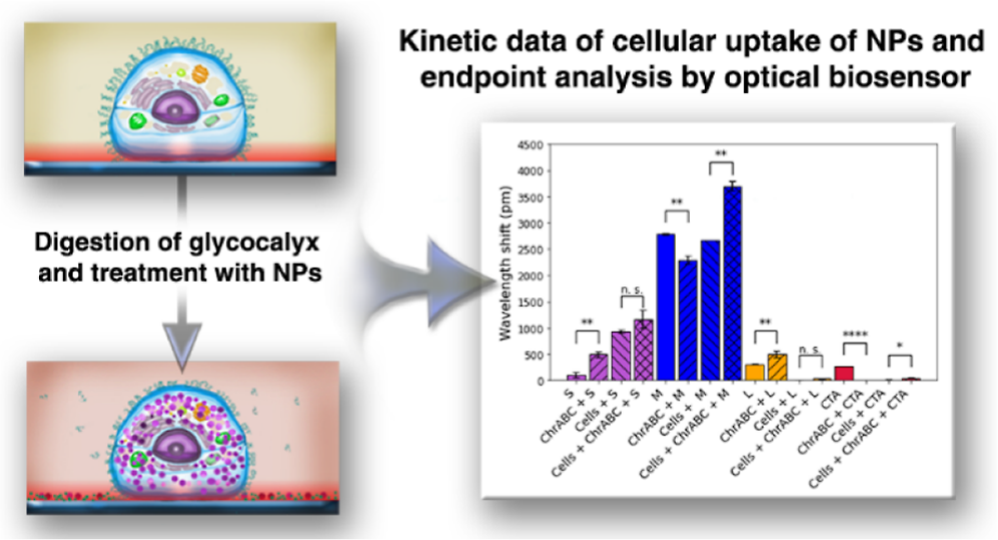

Functionalized nanoparticles (NPs) are widely used in targeted drug delivery and biomedical imaging due to their penetration into living cells. The outer coating of most cells is a sugar-rich layer of the cellular glycocalyx, presumably playing an important part in any uptake processes. However, the exact role of the cellular glycocalyx in NP uptake is still uncovered. Here, we in situ monitored the cellular uptake of gold NPs—functionalized with positively charged alkaline thiol (TMA)—into adhered cancer cells with or without preliminary glycocalyx digestion. Proteoglycan (PG) components of the glycocalyx were treated by the chondroitinase ABC enzyme. It acts on chondroitin 4-sulfate, chondroitin 6-sulfate, and dermatan sulfate and slowly on hyaluronate. The uptake measurements of HeLa cells were performed by applying a high-throughput label-free optical biosensor based on resonant waveguide gratings. The positively charged gold NPs were used with different sizes [*d* = 2.6, 4.2, and 7.0 nm, small (S), medium (M), and large(L), respectively]. Negatively charged citrate-capped tannic acid (CTA, *d* = 5.5 nm) NPs were also used in control experiments. Real-time biosensor data confirmed the cellular uptake of the functionalized NPs, which was visually proved by transmission electron microscopy. It was found that the enzymatic digestion facilitated the entry of the positively charged S- and M-sized NPs, being more pronounced for the M-sized. Other enzymes digesting different components of the glycocalyx were also employed, and the results were compared. Glycosaminoglycan digesting heparinase III treatment also increased, while glycoprotein and glycolipid modifying neuraminidase decreased the NP uptake by HeLa cells. This suggests that the sialic acid residues increase, while heparan sulfate decreases the uptake of positively charged NPs. Our results raise the hypothesis that cellular uptake of 2–4 nm positively charged NPs is facilitated by glycoprotein and glycolipid components of the glycocalyx but inhibited by PGs.

## Introduction

Functionalized nanoparticles (NPs) can be applied as cell-targeting drug carriers in biomedical applications.^1,2^ The penetration of NPs into cells has significant implications for medical treatments.^3−9^ NP interactions with a specific tissue cell significantly define its targeting capabilities and final therapeutic efficacy, where NP solubility and the stability of the delivered drug are also important factors to be considered.^2,10^ However, the real-time kinetics and dynamics of NP–living cell interactions are poorly investigated.

There are a lot of methods to investigate the cellular uptake of NPs by cells. For visualization, the following techniques can be applied, such as the confocal, fluorescence, dark-field, differential interference contrast microscopies, surface-enhanced Raman spectroscopy, and transmission electron microscopy (TEM).^11−18^ Inductively coupled plasma mass spectrometry (ICP–MS), ICP atomic emission spectroscopy, electron spin resonance, and fluorometry are demonstrated to measure the number of delivered NPs inside the cells.^11−13,19−25^ However, the most important drawback of these methods is that they provide end-point data, thus do not give information on the dynamics and kinetics of the uptake process.^26,27^ Nowadays, label-free biosensors and techniques receive more focus^28−40^ even in monitoring the real-time cellular uptake of NPs.^16,26,27^

In our previous work,^1^ we exploited an evanescent field-based optical biosensing technique to monitor the in situ cellular uptake of positively charged gold NPs (AuNPs).^1^ We revealed that the uptake of these functionalized NPs is an active process. Positively charged particles penetrated more effectively than negatively charged particles, and the optimal size for live cell uptake of the positively charged particles was measured to be around 5 nm. The fate of the NPs was further revealed by TEM on NP-exposed and subsequently fixed cells, well confirming the results obtained from the real-time kinetic data.^1^

The glycocalyx is a multifunctional cell surface sugar layer composed mainly of glycoproteins, glycolipids, and proteoglycans (PGs).^41^ The main backbone molecules of glycocalyx are plasma membrane (PM)-integrated glycoproteins and glycolipids, but it contains several membrane-associated proteoglycans (PGs) as well (i.e., syndecan and glypicans).^42^ PGs are composed of core protein and glycosaminoglycan (GAG) chains. Bound GAGs are polymers of straight-chain disaccharide monomers of various lengths that have been modified by sulfation or deacetylation, therefore carrying a negative charge at neutral pH. There are five types of GAGs: heparan sulfate (HS) and hyaluronic acid (HA), chondroitin sulfate (CS), keratin sulfate, and dermatan sulfate.^43,44^ Glycoproteins and glycolipids may be membrane integrated or secreted.^45^ These components are also decorated with sugar residues, but their oligosaccharide chains are often sialylated.^45^

The thickness of glycocalyx can be even hundreds of nanometers,^46,47^ and it carries pores formed by adjacent repulsive negatively charged sugar branches.^46^ Because of its strictly ordered 3D morphology^46^ and charge pattern, glycocalyx acts as a molecular sieve, preventing the passage of large molecules and restricting the access of certain molecules from the extracellular matrix to the cell membrane.^45^ Another function of the surface sugar coat is to regulate the microenvironment: GAGs can be very diverse due to epimerization, differences in length, and most importantly sulfation, which makes the surface of the glycocalyx extremely heterogeneous, making it suitable for docking many molecules.^44^ Glycocalyx serves as a signaling platform: its several transmembrane proteins may act as mechanotransducers because of their embeddedness in the submembranous actin network.^42^ It is able to dissipate the shear stress of the fluid from the membrane, making it responsible for shear-induced cellular processes such as cytoskeletal rearrangement.^36,44,48^

Glycocalyx components are supposed to take part in the regulation of receptor functions.^49^ It can also facilitate or inhibit the cellular uptake of growth factors, viruses, and functionalized NPs. Type and efficiency of uptake proved to depend on the NP size and coat, the intercellular medium composition, the cell type, and the cell line as well.^49^ Enzymatic removal of any component can significantly affect the functions and properties of the glycocalyx.^44^ The digestion of this sugar-rich coating of the cells is treated by the chondroitinase ABC enzyme (ChrABC) that catalyzes the eliminative degradation of GAG chains of the glycocalyx containing (1–4)-β-D-hexosaminyl and (1–3)-β-D-glucuronosyl or (1–3)-α-l-iduronosyl linkages to disaccharides containing 4-deoxy-β-D-gluc-4-enuronosyl groups.^36^ It acts on chondroitin 4-sulfate, chondroitin 6-sulfate, and dermatan sulfate and slowly on HA.^36^ The degree of digestion depends, of course, on the concentration of the enzyme, but even with an enzyme concentration of up to 135 mU/mL, most of the CS can be removed from the glycocalyx.^50^ Heparinase III (Hep III) cleaves the glycosidic bond between hexosamines and either iduronic acid or glucuronic acid residues. It contains 13 histidine residues, which are responsible for its catalytic action. It specifically degrades HS at the H_NY_–I and H_NY,6X_-G^2^ links.^51^ Neuraminidase (Neur) digestion eliminates the terminal sialic acids from the oligosaccharide chains of glycoproteins and glycolipids. It cleaves terminal sialic-acid residues that are α2,3-, α2,6-, or α2,8-linked to Gal, GlcNAc, GalNAc, AcNeu, GlcNeu, oligosaccharides, glycolipids, or glycoproteins.^52^

Removal of some components of the glycocalyx can cause changes in its 3D structure (pore size) and the shape of the cell membrane and can also affect the arrangement and activity of the receptors.^36^

In 2016, Cheng et al.^51^ studied 10 nm PEG-AuNP uptake through the robust glycocalyx of rat fat pad endothelial cells (RFPECs) by confocal microscopy. In their study, the HS chains of glycocalyx were digested. They found that AuNP uptake by enzymatic undigested endothelial cells was inhibited by glycocalyx because the pores of the glycocalyx were typically 7 nm wide. However, 6–7 times more AuNPs passed through the intracellular spaces through the dysfunctional enzymatic digested sugar layer, while some of the AuNPs were trapped in the degraded exogenous HS.^51^ In another study in the same year, Möckl et al.^53^ investigated the role of the glycocalyx component of the HS GAG in NP uptake. The uptake of 50 nm polystyrene NPs by human umbilical vein endothelial cells (HUVECs) was determined by confocal microscopy. They also found that the uptake of the polymer NPs was significantly increased with glycocalyx digestion but remained low in cells with native glycocalyx. This means that the endothelial glycocalyx is a barrier to the incorporation of NP into HUVECs.^53^ In 2021, Walter et al. studied the NP uptake of brain endothelial cells by zeta potential measurement. Neur digestion of sialic acid residues of blood–brain-barrier (BBB) endothelial surface glycocalyx was found to increase cellular uptake of glutathione and alanine dual-targeting NPs compared to untreated cells.^54^ Authors draw attention that the glycocalyx of BBB endothelial cells has special features, and its thickness, composition, and permeability differ in the central nervous system and periphery.

Most cancer cells have severely thickened and altered-composition glycocalyx.^36,55^ Abundantly secreted HA contributes to drug resistance in breast cancer. The antitumor activity of NPs loaded with HA self-assembling oligosaccharide chains was determined. The results showed that heparinase can break down the cell envelope associated with cells, making breast cancer cells more vulnerable. The use of these NPs reduced tumor growth in a mouse model carrying breast cancer.^56^

Here, we investigate the role of glycocalyx components in NP cellular uptake. Gold NPs with different sizes and charges were used in live cell experiments with an evanescent-field-based label-free optical biosensor. In order to investigate the bare NP–living cell interactions and to avoid any protein corona effects,^57−59^ the experiments were run in protein-free buffer solutions. Real-time kinetic curves of the uptake of NPs were recorded with various components of the glycocalyx removed by enzymatic treatment. The mechanism of uptake was investigated by ultrastructural localization of internalized gold NPs.

## Materials and Methods

### Cell Culture and Cell Adhesion Assay Buffer

HeLa cells (ECACC 93021013 human, cervix, epitheloid, and carcinoma) were maintained in a humidified incubator (37 °C, 5% CO_2_) in Dulbecco’s modified Eagle’s medium, supplemented with 4 mM L-glutamine, 10% fetal bovine serum (Biowest SAS, France), 100 U/mL penicillin, 100 μg/mL streptomycin solution, and 0.25 μg/mL amphotericin B. Cell adhesion assay buffer was prepared by adding 20 mM 4-(2-hydroxyethyl)-1-piperazine ethanesulfonic acid (HEPES, Sigma-Aldrich) to Hank’s balanced salt solution (HBSS, Sigma-Aldrich), pH 7.0.^1^

### Preparation of Positively and Negatively Charged AuNPs

To study the influence of NP size on the cellular uptake, we synthesized various sizes of positively charged AuNPs (*d* = 2.6 ± 0.6, 4.2 ± 0.7, 5.4 ± 1.3, and 7.0 ± 1.2 nm). In this case, the surfaces of AuNPs were coated with (11-mercaptoundecyl)-*N*,*N*,*N*-trimethylammonium bromide (TMA) according to a modified literature procedure.^1^ Briefly, presynthesized AuNPs were mixed with an excess amount of TMA dissolved in dichloromethane. The mixture containing AuNPs and TMA was stirred for 1 h to bind TMA to the surface of AuNPs. Thus, prepared TMA-coated AuNPs (henceforth, AuTMA) were purified with a solution of toluene, methanol, and dichloromethane using a rotary evaporator. Last, AuTMA was homogeneously dispersed in ultrapure water at a concentration of 15 mM (gold atoms). This dispersion was used as a stock solution of AuTMA. The average diameter of AuNPs was determined by using TEM (JEM-2100, JEOL). The concentration of AuTMA (in terms of gold atoms) was determined from their maximum absorption (near 520 nm) using an ultraviolet–visible spectrophotometer. Later, this stock solution was diluted with HBSS–HEPES to 5.00, 0.50, and 0.05 mM (gold atoms). Citrate-capped (negatively charged) AuNPs (*d* = 5.5 ± 0.6 nm) were synthesized by a modified citrate reduction method using tannic acid described in detail in our previous work.^1^ We carried out dynamic light scattering (DLS) and zeta potential measurements (Malvern Zetasizer) in the HBSS–HEPES buffer. We found that TMA-functionalized NPs were stable in the buffer after 24 h (we observed no change in the size and their distribution). In the case of citrate-stabilized NPs, in the buffer solution after 24 h, the average size changed from 7 to 23 nm, indicating a slight aggregation. However, in all cases, the magnitude of the zeta potential was in the range between 20 and 30 mV.

### Transmission Electron Microscopy

**Figure 1:**
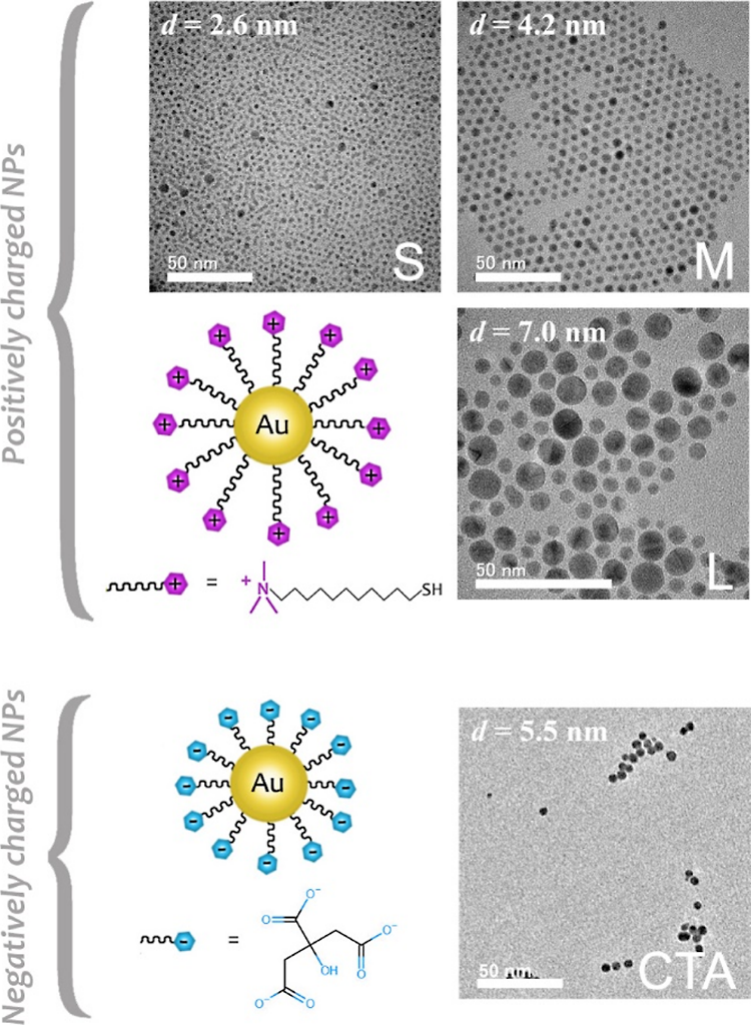
Schematic illustration of the positively charged gold NPs functionalized with TMA (purple, upper side) and the negatively charged citrate-capped tannic acid (CTA, blue, bottom side) AuNPs. The TEM micrographs show the AuTMA NPs in four different sizes (S, M, and L) and a TEM micrograph of the CTA NPs.

The NP solutions were drop-dried on carbon-coated microgrids for TEM measurements. A Thermo Fisher Scientific THEMIS 200 aberration-corrected microscope was used for taking overview images and determining the size distribution as well as for high-resolution images. The TEM resolution of the TEM/scanning TEM unit is 0.07 nm, and the images were recorded with a CETA 16-megapixel camera. The TEM micrographs and the schematic illustrations of the NPs are shown in Figure 1.

Cell fixation and sample preparation for ultrastructural localization of internalized AuNPs were described earlier.^1^ Briefly, after AuNP treatment, HeLa cells were trypsinized and washed and then were fixed for 24 h at 4 °C (3.2% paraformaldehyde, 0.2% glutaraldehyde, 40 mM CaCl_2_, and 1.0% sucrose in 0.1 M cacodylate buffer, pH 7.2). Treatment with 1% osmium tetroxide (1 h at room temperature) was followed by counterstaining with 1% aqueous uranyl acetate (30 min). Dehydration was carried out with ascending grades of alcohol and absolute ethanol. Finally, samples were embedded in Spurr low-viscosity epoxy resin (Sigma-Aldrich). Ultrathin sections were stained with 2.5% aqueous uranyl acetate (10 min) and Reynolds’s lead citrate (3 min) and were examined using a JEOL JEM 1011 transmission electron microscope. Micrographs were taken using an 11-megapixel Morada camera with the aid of iTEM software (Olympus).

### Resonant Waveguide Grating Optical Biosensor

We employed the Epic Benchtop (BT) system (Corning Incorporated, Corning, NY, USA) resonant waveguide grating (RWG) label-free optical biosensor. The Epic BT device can operate with 96- or 384-well standard Society for Biomolecular Screening (SBS) format cell assay microplates. This label-free setup allows the sensors to be addressable individually by having a separate planar optical waveguide (made of niobium pentoxide) equipped with a 2 × 2 mm optical grating at the bottom of each well. The optical gratings interrogate the TM0 waveguide mode with near-infrared electromagnetic radiation. The Epic BT measures the resonant wavelength (λ) of each sensor every 3 s by tuning the incoupled wavelength in the range of 825–840 nm with a 0.25 pm precision. An optical waveguide enters an excited state on its resonant wavelength, in which any difference of the refractive index (RI) in the ∼150 nm thick evanescent field above the surface of a sensor alters the resonant wavelength to a λ′-shifted resonant wavelength. As every biomolecule has an RI larger than that of water, their presence in the probing depth above the sensor surface causes RI changes. Processes such as dynamic cell mass redistributions, cellular spreading, or biomolecular surface adsorption can all lead to measurable RI variations. The Epic BT records the resonant wavelength shift (Δλ = λ′—λ) which is the difference between the shifted resonant wavelength (λ′) and the resonant wavelength of the optical waveguide (λ).^1^

### Label-free Optical Biosensor Measurements

**Figure 2:**
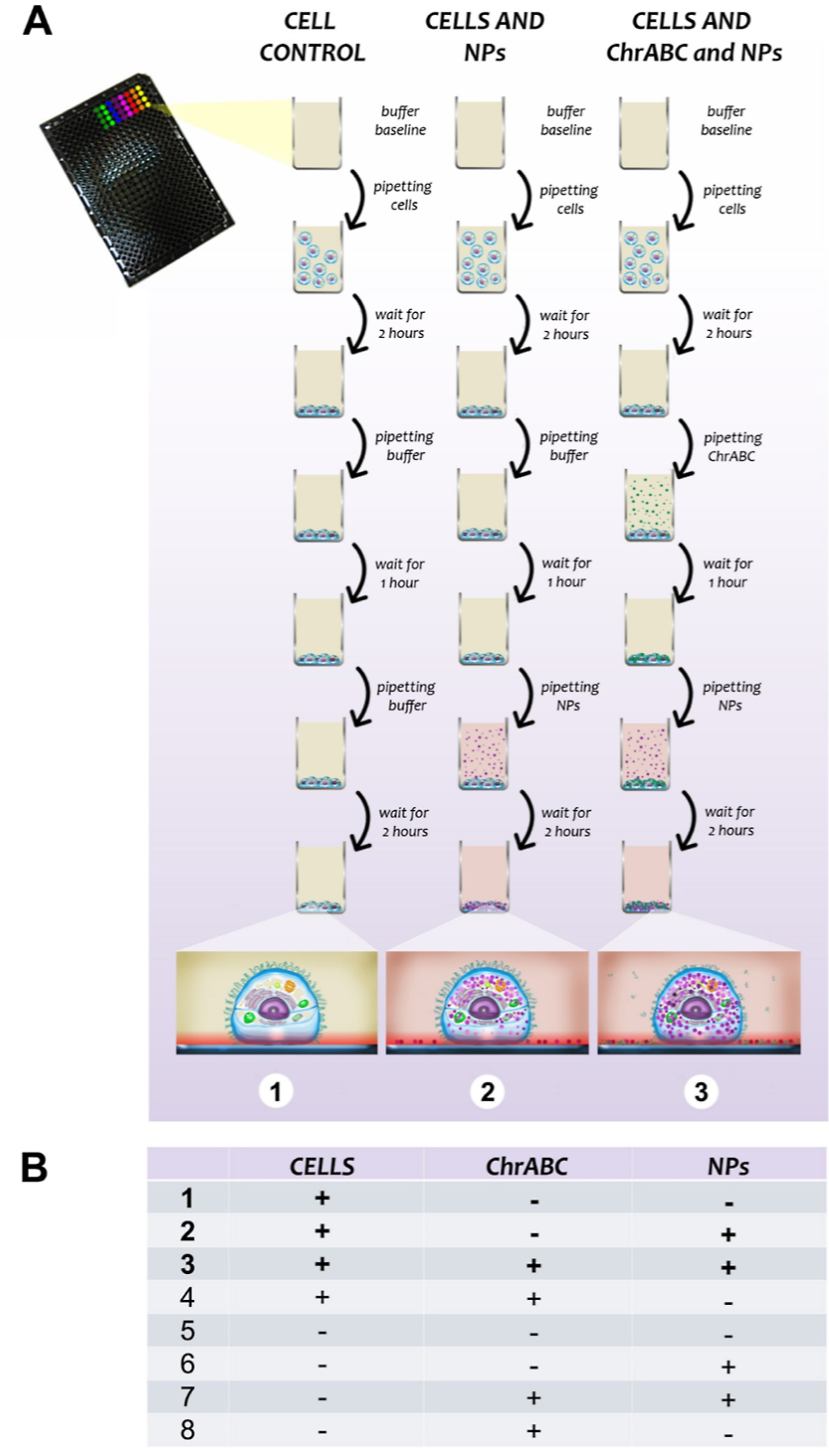
Protocol of the biosensor measurements. A. Measurement steps in case of the cell control (1), cells and NPs (2), and cells and ChrABC and NP measurements (3). Due to the plate format, simultaneous and parallel experiments can be performed. B. Table of the measurement options. The no. 1, 2, and 3 options are illustrated in the “A” part. The ,,+” means the presence and ,,-” means the lack of cells, ChrABC, or NPs.

After filling the wells (in a 384-well plate) with HBSS–HEPES (30 μL), the baseline was taken by the Corning Epic appliance for 40–60 min. Then, we paused the baseline and removed the buffer. The HeLa cells were detached with trypsin–ethylenediaminetetraacetic acid solution. Cells were centrifuged for 5 min at 200*g* and resuspended in the employed assay buffer. The cells were counted in a hemocytometer, and 20,000 cells were pipetted to the wells with 20 μL of the assay buffer inside and we also added 20 μL of buffer into the control wells. We measured them for 2 h. Later, 20 μL of ChrABC solutions (0.6 U/mL) or buffer (to control wells) was pipetted into the wells, and we measured the cell adhesion with the ChrABC for a further 1 h. After that, we pipetted the solutions of NPs or the buffer to wells (20 μL). The AuNPs were measured for further 2 h. All measurements were done in four parallel experiments. The schematic illustration of the measurement steps is shown in Figure 2.

### Optical Microscopy of the Cells

At the end of the Epic BT biosensor experiments, the microplate was put into a Zeiss Axiovert Observer microscope to image the cells and the aggregated NPs on their surfaces.

### Statistics and Data Processing

RWG measurement data are plotted as means ± standard deviation. Statistical significance between treatment groups was calculated using one-way ANOVA. The number of parallel samples was at least three. Changes were statistically significant at *p* < 0.05.

## Results and Discussion

The employed RWG sensor monitors RI variations in the close vicinity of the sensor surface, within a depth of around 100–200 nm. Gold NPs have larger polarizability than water; therefore, particles entering the evanescent field increase the local RI and shift the monitored resonant wavelength.^1^

**Figure 3:**
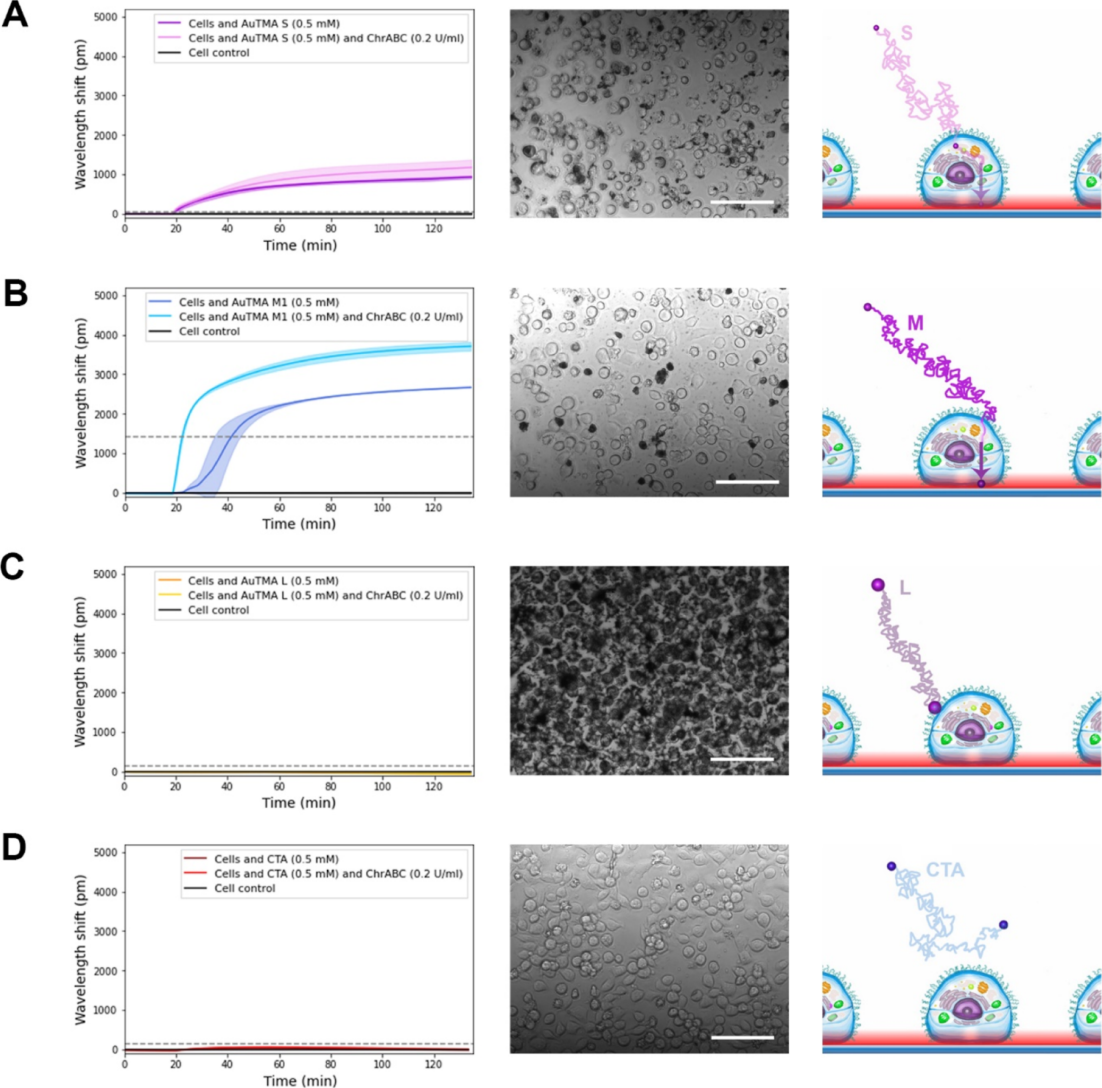
Kinetics of the S-, M-, L-sized positively charged and the negatively charged CTA AuNP gold NP’s cellular uptake. The dashed line in every graph represents the half value of the NP adsorption endpoint data without cells being present on the surface. A. Kinetic curves of S AuNPs. Glycocalyx digestion a little bit increased the particle uptake of the cells. In the middle, microscopic images of the cells and S NPs in the biosensor well after the measurement are shown. On the right, the pathway of a single S-sized particle is illustrated. The NP reaches the evanescent field, and thus the biosensor senses the alteration in the cell. B. Kinetic curves of M AuNPs. Glycocalyx digestion drastically increased the particle uptake of the cells. In the middle, microscopic images of the cells and M NPs in the biosensor well after the measurement are shown. On the right, the pathway of a single M-sized particle is illustrated. The NP reaches the evanescent field inside the cell, and thus the biosensor senses the alteration in the cell. C. Kinetic curves of L AuNPs. According to the kinetic curves, the L NPs do not penetrate the cells. Glycocalyx digestion has not got an effect on this process at all. In the middle, microscopic images of the cells and L NPs in the biosensor well after the measurement are shown. On the right, the pathway of a single L-sized particle is illustrated. The NPs do not reach the evanescent optical field, meaning no change in the recorded biosensor signal. If the L-sized NP is added to the biosensor without a cell, it will adhere to the empty surface, but if cells are added, since the signal has not changed, the NP may not reach the surface, but it cannot penetrate the cell, it cannot absorb by endocytosis. It will most likely stick to the surface of the cell. D. Kinetic curves of negatively charged CTA AuNPs. According to the kinetic curves, the CTA AuNPs do not penetrate the cells. Glycocalyx digestion has not got an effect on this process at all. In the middle, microscopic images of the cells and CTA NPs in the biosensor well after the measurement are shown. On the right, the pathway of a single CTA particle is illustrated. The NPs reach only the bare biosensor surface or reach only the surface of the cells, so there is no biosensor signal. The scalebar is 100 μm.

The recorded time-dependent resonant wavelength shift signals are shown in Figure 3 when cells are present at 50% confluency on the surfaces, and NPs with various sizes and surface charges are added to the adhered cells. When the added NPs enter the evanescent optical field, they increase the wavelength shift signal as clearly seen in Figure 3A,B (positively charged particles with sizes of 2.6 and 4.2 nm).

NP can enter the evanescent field by two independent mechanisms: (i) simple adsorption on the surface of the waveguide at the bare, cell uncovered areas and (ii) cellular uptake and transfer of particles into the evanescent field inside the adhering cells. In order to decide between the above-mentioned possibilities, we measured the adsorption of the NPs on bare sensor surfaces without the adhering cells. We hypothesize that the NPs do not interact with the adhering cells. In this case, the signal recorded with the cells should be exactly 50% of the signal measured on the bare surfaces due to the reduction to 50% of the free available bare sensor area. The 50% of the bare sensor signals are shown as dashed lines in Figure 3 in all cases investigated. Clearly, any value above or below the dashed lines means an interaction between the cells and the NPs. This strategy, therefore, gives a convenient way to monitor the interactions of NPs and living cells, and the effect of enzymatic digestion of the glycocalyx component can also be investigated (see Figure 3).

It should be noted that the thickness of the glycocalyx varies from a few nanometers to even 3 μm, depending on the cell type,^60^ and there are significant differences in surface charge values between various cell lines depending on their function, origin, and presumably glycocalyx composition.^36^ In our previous work, we measured the changes of HeLa cell glycocalyx degradation after ChrABC enzyme treatment by zeta potential and CS immunostaining.^36^ The basal zeta potential of HeLa cells was –11.9 mV. ChrABC treatment significantly changed the zeta potential after 60 min treatment (1.25 U/ml) to – 10.2 mV, as a consequence of the removal of negatively charged CS. The CS-staining measurements showed that there is a decrease (approximately 33%) in the fluorescence intensity at and above the 1.25 × 10^–3^ U/ml ChrABC concentration, indicating the degradation of the glycocalyx in case of 60 min incubation time. ChrABC treatment reduces the CS amount on HeLa cells but leaves other components less affected.^36^

From the monitored kinetic data, we conclude that the positively charged S (2.6 nm)- and M-sized (4.2 nm) NPs (0.5 mM) were taken up by the cells, and the uptake was facilitated by the ChrABC treatment. See the larger signals and/or different, higher slopes in Figure 3A,B. It is important to note in case of M-sized (4.2 nm) AuNPs, the penetration after glycocalyx treatment is more pronounced. The addition of ChrABC significantly accelerates the entry of M-sized AuNPs (compare Figure 3A,B).

In contrast, L-sized NPs (*d* = 7.0 nm, 0.5 mM) do not enter the evanescent optical field when cells are present on the surface. The signal level is well below the dashed line, meaning that the NPs do not reach the bare sensor areas. Based on the recorded optical micrographs (see the right column in Figure 3), we assume that the L-sized NPs are effectively adsorbing on the surface of the cells. Based on the biosensor data, it can also be safely concluded that these adsorbed NPs do not reach the evanescent optical field. Either the NPs are not taken up by the cells or there is another unknown effect that effectively blocks the transfer of uptaken NPs to reach the evanescent optical field. Presumably, the internalization of the 7.0 nm particles is less effective. This possibility is also supported by literature data.^1,61^ Our present results show that the ChrABC treatment had no effect and does not increase the signal of L-sized NPs (see Figure 3C). According to these results, the diameter of around 4 nm seems to be the optimal size for NP uptake, and up to this size, ChrABC treatment facilitates the cellular uptake of positively charged particles.

In a control experiment, negatively charged NPs [0.5 mM citrate-stabilized AuNPs (CTA)] were also investigated. Our results show that the cells did not interact with these particles and the enzyme treatment did not change this behavior (see Figure 3D). The drawings in the middle column of Figure 3 schematically show the obtained interactions in the various cases.

**Figure 4:**
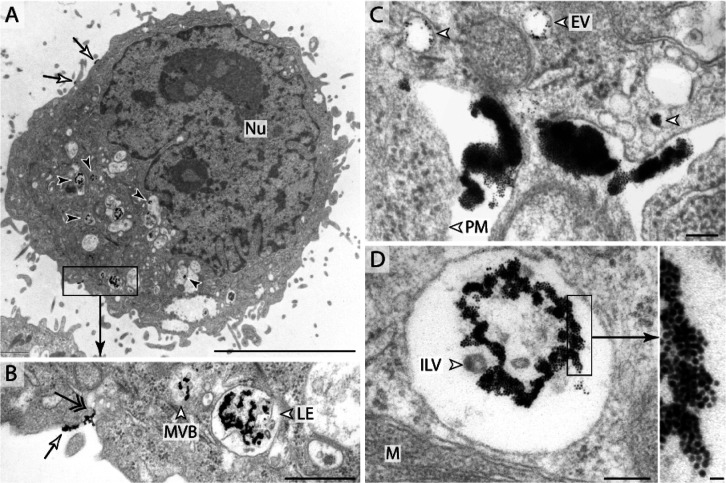
Uptake of M-sized positively charged NPs in HeLa cells. (A) Overview of a cell with NPs on the cell surface (arrows) and in the endolysosomal compartment (black arrows). (B) At higher magnification, AuNPs can be detected on the PM (arrow) during internalization (double-headed arrow) in the MVB and LE. (C) NPs on the PM and early EVs (white arrowheads). (D) Accumulated AuNPs in LE containing intraluminal vesicles. Inset: high-magnification gold NPs (scale bars: A—5 μm, B—500 nm, C, D—100 nm, and D inset—10 nm).

Ultrastructural localization of M-sized AuNP was further investigated by TEM to show and prove that the M-sized NPs penetrate the cells according to the kinetic data with the highest efficacy (Figure 3B). Internalization is energy-dependent endocytosis when the NP accumulation is detected only in membrane-bordered compartments of the endo-lysosomal system. The entering mechanism is passive, if NPs appear exclusively (or mainly) in the cytosol and nucleus. In the case of 4.6 nm TMA-coated gold NPs, the uptake mechanism proved to be active endocytosis: NPs were identified on the cell surface in early endosomal vesicles (EVs), in multivesicular bodies (MVBs), and late endosomes (LEs) (Figure 4). Internalization was a rapid process due to the fact that NP accumulation was detected mainly in the late endosomal compartments. Other cell organs (endoplasmic reticulum, Golgi stacks, and mitochondria), cytoplasm, and nucleus were negative. These results indicate that treatments did not damage membrane integrity and functions.

**Figure 5:**
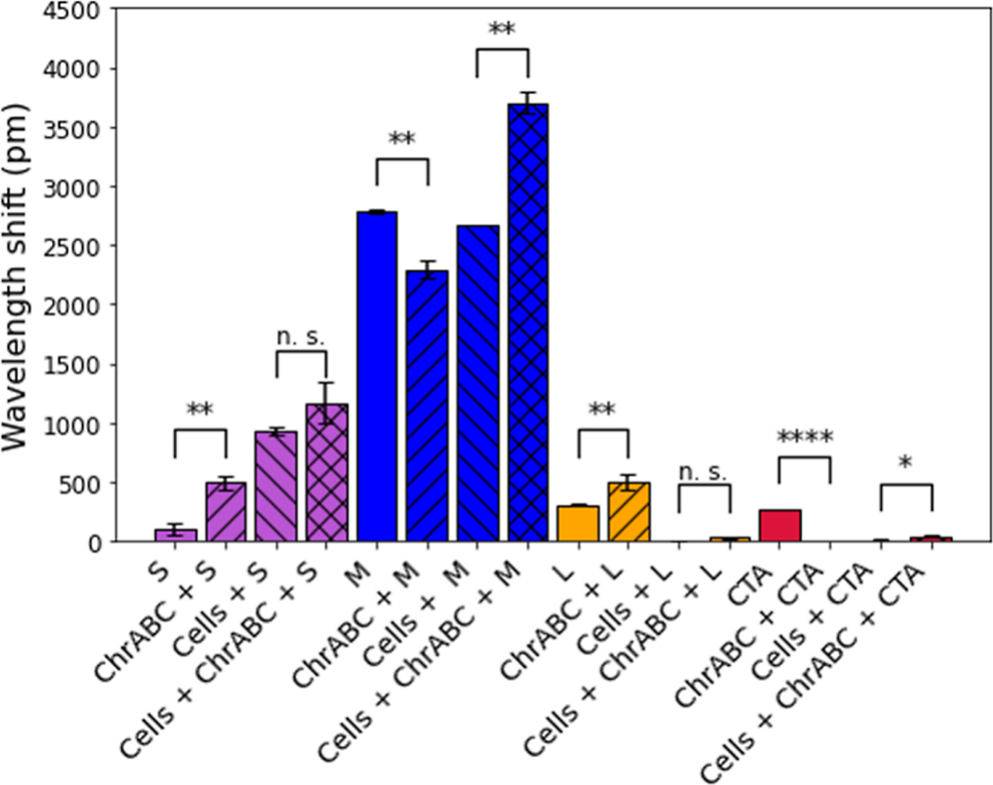
Plotted endpoint data of the measurement curves. Endpoint data of adsorption kinetic curves in case of NPs (S, M, L, and CTA) and NPs together with the ChrABC enzyme on a bare biosensor surface. Endpoint data of the adhesion kinetic curves of the treated cells with NPs and NPs together with the ChrABC enzyme are also plotted in the case of all types of NPs. The kinetic endpoint values are corrected with their controls. *p < 0.05, **p < 0.01, ***p < 0.001, and ****p < 0.0001.

For comparison, the endpoint signals of the various measurements are summarized in Figure 5. Experiments, in which ChrABC was added to the NPs without adhering cells, were also performed, and the results are shown in Figure 5, too. Enzyme addition slightly changed the bare sensor signals but did not affect the above conclusions about the cell–NP interactions.

In additional pilot experiments, other enzymes were also tested to investigate their impact on NP cellular uptake. The best-performing M-sized AuTMA NPs were used in these additional measurements in 0.5 mM Au concentration. The applied enzymes were the ChrABC, Hep III, and Neur in two different concentrations (1.25 or 0.125 U/mL and 3.33 or 0.333 mU/mL in the case of HepIII). ChrABC acts on chondroitin 4-sulfate, chondroitin 6-sulfate, and dermatan sulfate and slowly on HA of the glycocalyx. Hep III contains 13 histidine residues, which are responsible for its catalytic action. It specifically degrades HS at the H_NY_–I and H_NY,6X_-G^2^ links.^51^ Neur cleaves terminal sialic acid residues that are α2,3-, α2,6-, or α2,8-linked to Gal, GlcNAc, GalNAc, AcNeu, GlcNeu, oligosaccharides, glycolipids, or glycoproteins.^52^ Zeng et al. showed the effects of enzymatic digestion in the case of ChrABC and Hep III.^62^ The authors used RFPECs, and the incubation time was 2 h. There were no changes in the coverage, average, and junctional thickness of HA after Hep III treatment. However, Hep III treatment degraded HS; the coverage changed from 83,3 to 2.6%, the average thickness decreased by 54.9%, and junctional thickness decreased by 55% after 3645 mU/ml treatment. In the case of RFPECs, the initial coverage of CS decreased by 16.5%, the average thickness was significantly decreased by 31.3%, and the junctional thickness also significantly decreased by 42.8% in the presence of 405 mU/mL ChrABC.^62^ Gentsch et al. showed that under the Neur digestion conditions (10 mU/ml), the authors were able to remove a maximum of 75% of the total N-acetylneuraminic acid of L929 murine cells (and thus, the number of receptor sites for type-3 reovirus was also reduced by 47%).^63^

**Figure 6:**
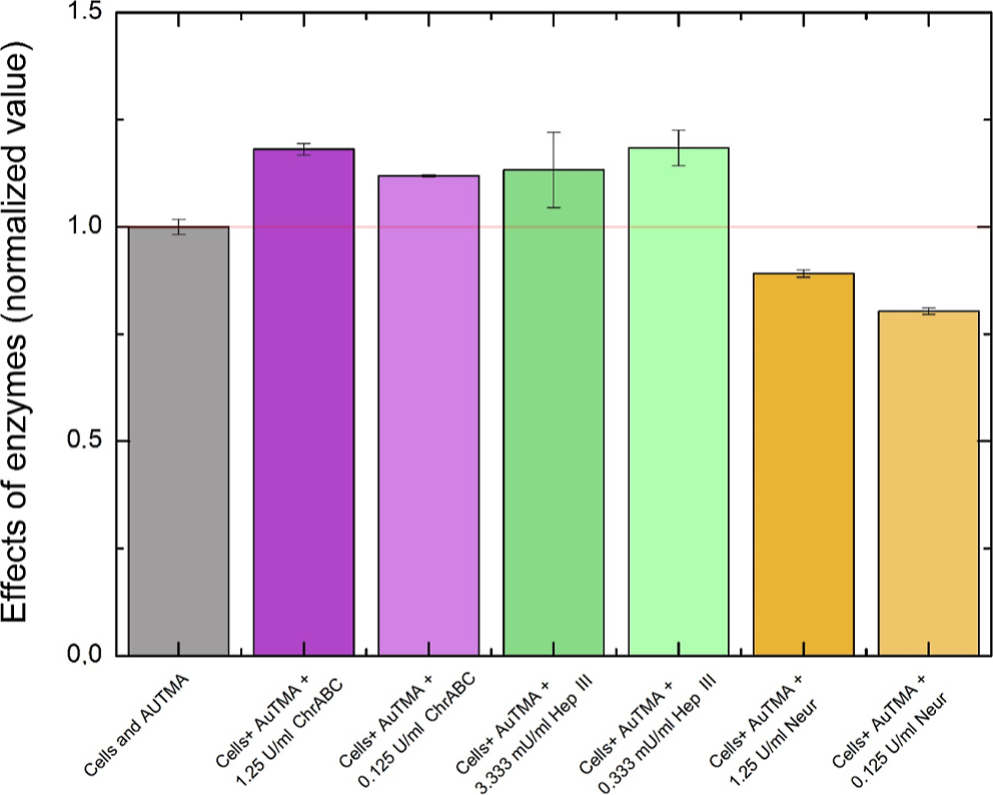
Normalized RWG endpoint data of NP uptake by the cells with different types of enzymes (ChrABC, Hep III, and Neur) in different concentrations. ChrABC and Hep III increase, while Neur decreases the cellular uptake of NPs compared to the enzyme-free control.

The normalized endpoint data of the measurements are plotted in Figure 6. The results show that ChrABC and Hep III are the most effective enzymes in facilitating the uptake of positively charged NPs. In contrast, Neur treatment decreases the cellular uptake of positively charged NPs. Interestingly, the larger concentration has less effect in several cases suggesting an optimal glycocalyx structure for cellular uptake of NPs. However, the explanation of the observed phenomena and the magnitude of the measured changes need further investigation outside of the scope of the present study.

Several experimental data confirm that efficacy of NP uptake also depends on the features of the gold NP (size, charge, and coating) and the cell type (glycocalyx). Enzyme treatment can modify the parameters of the internalization through alteration of the physical and chemical structure of the glycocalyx. Uptake can be stimulated if the glycocalyx thickness decreases, pore size increases, or eliminated components have any inhibitory effect on internalization (i.e., bound them before NPs reach the PM). However, reduced internalization upon enzymatic treatment indicates the important role of eliminated components in the uptake.^49^

In our experimental system, GAG-digested ChrABC and Hep III treatment increased TMA-NP uptake of HeLa cells in case of S- and M-sized particles. Similar results have been published in the case of 10 nm PEG-AuNP by Cheng et al.^51^ and 50 nm polystyrene nanospheres by Mökl et al.^53^ after digesting HS chains of the endothelial glycocalyx. In our experiments, enzymatic digestion takes 1 h before adding gold NPs and 2 h in the presence of AuNPs. The enzymatic reaction was not terminated; therefore, during the entire period, newly synthetized glycocalyx components were also destroyed. Enzyme concentration and treatment time were determined earlier considering the maintenance of cell viability and membrane integrity.^36,43^ The supernatant was not eliminated before the addition of NPs during the biosensor measurement, but in our opinion, the rapidly dispersed liberated fragments had no significant effect on NP uptake. Although most data support a positive role of HS in internalization,^49^ our results raised the hypothesis that this glycocalyx component has an inhibitory role in cellular uptake of S- and M-sized TMA-coated AuNPs. This inhibitory effect shows an inverse correlation with the size of NPs.

In the case of Neur, we detected decreased cellular NP uptake upon enzymatic digestion. This result indicates the involvement of sialylated glycoproteins and glycolipids in the internalization. In the case of endothelial BBB, the Neur treatment stimulated the uptake of glutathione and alanine dual-targeting NPs.^54^ Both the glutathione and alanine have a Na-coupled carrier-mediated transport on the luminal surface of blood vessels.^54,64^ Carriers are specific receptors of the PM, recognize their target molecules, and actively transfer them to the other side of the membrane. Increasing uptake indicates that desialylation of glycocalyx did not destroy these carrier functions, moreover, helped NPs to reach the PM. In the view of enzymatic treatment consequences, this mechanism differs from our results. Because desialylation inhibited the internalization, we hypothesize that sialic acids of glycoproteins and glycolipids play role in the uptake of S- and M-sized TMA-NPs. Influenza viruses do not need specific receptors with internalization motifs: binding of virus hemagglutinin to sialic acid components of glycocalyx initiates virus endocytosis.^65^ After Neur treatment of host cells, virus internalization would be reduced, as in our AuNP experiments. Influenza viruses may also use clathrin-dependent and -independent pathways to enter the cells.^65^ Determination of the exact mechanism of TMA-AuNP internalization needs further examinations.

Earlier, in the same experimental system, we documented that 9.4 nm sized AuNPs were also internalized by HeLa cells, but their internalization rate was the lowest in comparison with 2.5 and 4.6 nm particles.^1^ However, we found that 7 nm L-sized NPs did not enter the cells, and any enzymatic treatment was unable to initiate the uptake. Wide intervallum (2–110 nm) was tested for effective cellular uptake of AuNPs (see Shang’s comprehensive review^66^). Since gold NP coating, tested cell types and measurement methods were diverse; therefore, the evaluation of our L-sized NP result is doubtful. Clarifying the background makes further investigations necessary.

## Conclusions

In this work, we monitored the penetration of different-sized (*d* = 2.6, 4.2, and 7.0 nm) TMA-functionalized, positively charged AuNPs into surface-adhered HeLa cells by employing a high-throughput resonant waveguide grating biosensor. We studied the role of the glycocalyx in the penetration of NPs by digesting various components of the PG layer of the cells prior the addition of NPs. Due to the applied plate-based format, numerous measurements could be carried out simultaneously (as clearly seen in Figure 1). The kinetics of the biological processes could also be analyzed cost-effectively. This is a clear advantage over large-scale instrumentations capable of endpoint measurements of fixed cells, such as electron microscopy. The shape and magnitude of the recorded kinetic data suggested that the S- and M-sized positively charged AuNPs are internalized into the cells and glycocalyx digestion by ChrABC facilitates their entry. However, larger particles (7 nm) could not enter the cells and even the ChrABC treatment does not effect it either. As we studied earlier, the native cell membrane is negatively charged; thus, an attractive electrostatic interaction can help the positively charged NPs to be internalized into the cells. NPs must first pass through the thick glycocalyx layer of cancer cells. It is likely that the initial binding of AuNPs to cells, in which the modified cell surface sugar layer has a significant effect, may affect the efficiency of endocytosis. Under the action of chondroitinase and heparinase, the glycocalyx barrier layer of the cells will be less intact, allowing the cationic NPs to reach the PM more rapidly, thereby enhancing endocytosis. However, the opposite effect in the case of Neur indicates the supporting involvement of sialylated glycoproteins and glycolipids in 2–5 nm sized TMA-Au NP internalization.

We found that the cellular uptake of negatively charged AuNPs, the CTA-capped ones, is far less effective, and the enzyme treatment did not have a significant impact on the process. According to our measurements with different enzymes, ChrABC is the most effective in facilitating the penetration of AuNPs into HeLa cells, and Neur treatment inhibits the NP uptake process.

Our finding supports the hypothesis that the cell membrane and even the PG components of the glycocalyx play a crucial role in the penetration of the positively charged NPs. The effect can be fine-tuned by enzymatic digestion of the glycocalyx which can find applications in cancer treatment and targeted drug delivery. The enzymatic removal can be a kind of additional treatment in cancer therapy, facilitating the efficiency of more conventional treatments. For instance, CS released by breast cancer cells plays a crucial role in the interaction with structural proteins, and it also promotes the development of metastasis.^67,68^

Lung cancer cells from mice were examined during ex vivo experiments.^69^ The employed ChrABC inhibited cell metastasis of lung cancer by removing CS, thereby reducing the number of nodules formed, so the metastasis in the lungs was effectively inhibited by the enzymatic removal of CS.^69^ The CS PG is required for the integrity of normal tissue structure, hence the targeted effects of ChrABC should be minimized, thus future therapeutic purposes may require a carrier for transporting the enzyme to the desired location.^67^ Thus, our present basic research experiments and the used method may help to design carrier molecules to transport enzymes to the target cells and cure different illnesses. This work may help to create tools for future therapies and medication with increased specificity for particular cell types.

## Supporting Information Available

Size distribution of the TMA-functionalized and CTA AuNPs of various sizes obtained by DLS measurements (PDF)
