# Supplementary material for: Glycocalyx Components Detune the Cellular Uptake of Gold Nanoparticles in a Size- and Charge-Dependent Manner
Source: ACS Appl Bio Mater. 2022 Oct 14;6(1):64–73. doi: 10.1021/acsabm.2c00595 (PMC9846697; doi:10.1021/acsabm.2c00595)
Supplement: Supplementary file 1 — mt2c00595_si_001.pdf [file mt2c00595_si_001.pdf]

Supporting Information

Glycocalyx components detune the cellular uptake of gold nanoparticles in a size- and charge-dependent manner

Beatrix Peter[a], Nicolett Kanyo[a]*, Kinga Dora Kovacs[a,b], Viktor Kovács[a], Inna Szekacs[a], Béla Pécz[c], Kinga Molnár[d], Hideyuki Nakanishi[e], Istvan Lagzi[,f,g], Robert Horvath [a]*

[a] *Nanobiosensorics Group, Institute of Technical Physics and Materials Science, Centre for Energy Research, Konkoly-Thege út 29-33, H-1120 Budapest, Hungary*

[b] *Department of Biological Physics, Eötvös University, Budapest, Hungary*

[c] *Thin Films Laboratory, Institute of Technical Physics and Materials Science, Centre for Energy Research, Konkoly-Thege út 29-33, H-1120 Budapest, Hungary*

[d] *Department of Anatomy, Cell and Developmental Biology, ELTE, Eötvös Loránd University, Pázmány Péter stny. 1/C, Budapest, H-1117, Hungary*

[e] *Department of Macromolecular Science and Engineering, Graduate School of Science and Technology, Kyoto Institute of Technology, Matsugasaki, Kyoto 606-8585, Japan*

[f] *Department of Physics, Institute of Physics, Budapest University of Technology and Economics, Műegyetem rkp. 3, Budapest H-1111, Hungary*

[g] *ELKH BME Condensed Matter Research Group, Műegyetem rkp. 3, Budapest H-1111, Hungary*

**equal contributions*

**e-mail: peter.beatrix@ek-cer.hu*

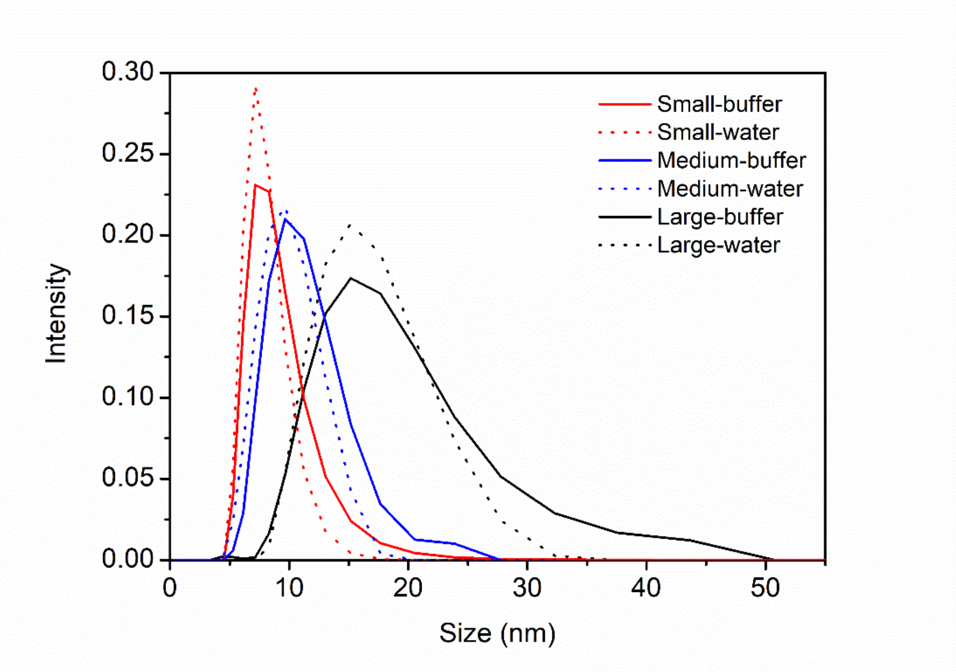

***Figure S1** The size distribution of the TMA functionalized AuNPs of various sizes obtained by DLS measurements in water (dotted line) and HBSS-HEPES buffer (solid line) after 24 hours. The composition of the HBSS-HEPES buffer: K^+, Na^+, Mg^{2+}, Ca^{2+}, Cl^-, HPO_4^{2-} /$H_2PO_4^-$ ions (all together 155 mM, mainly NaCl) and 2 mM glucose.*

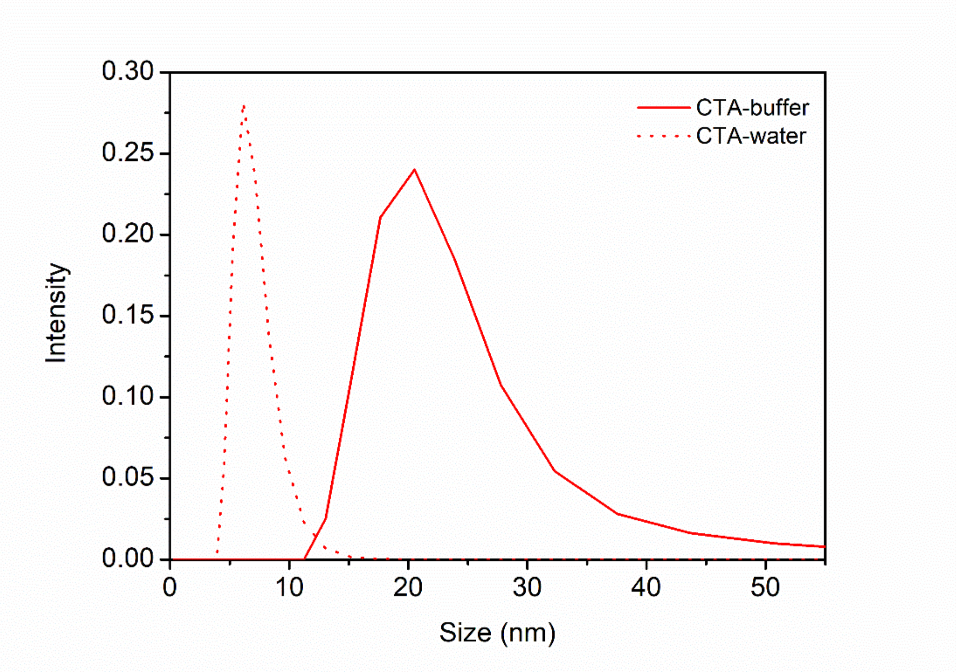

Figure S2 *The size distribution of citrate capped tannic acid AuNPs (CTA) obtained by DLS measurements in water and HBSS-HEPES buffer after 24 hours. The composition of the HBSS-HEPES buffer:* K^+, Na^+, Mg^{2+}, Ca^{2+}, Cl^-, HPO_4^{2-} /$H_2PO_4^-$ *ions (all together 155 mM, mainly NaCl) and 2 mM glucose.*

Table S1 *The average size (measured by TEM and DLS) and zeta potential of NPs in water and HBSS-HEPES buffer after 24 hours. The composition of the HBSS-HEPES buffer:* K^+, Na^+, Mg^{2+}, Ca^{2+}, Cl^-, HPO_4^{2-} /$H_2PO_4^-$ *ions (all together 155 mM, mainly NaCl) and 2 mM glucose.*

	Small NPs functionalized with TMA	Medium NPs functionalized with TMA	Large NPs functionalized with TMA	NPs functionalized with citrate
Average size (TEM)	2.4 nm	4.2 nm	7.0 nm	5.5 nm
Average size (DLS)	8.7 nm	10.9 nm	17.7 nm	6.8 nm
Average size (DLS) in HBSS-HEPES buffer	7.8 nm	9.6 nm	16.2 nm	23.0 nm
Average zeta potential in HBSS-HEPES buffer	31.5 mV	20.2 mV	23.8 mV	−34.2 mV
